# The contribution of Kv2.2‐mediated currents decreases during the postnatal development of mouse dorsal root ganglion neurons

**DOI:** 10.14814/phy2.12731

**Published:** 2016-03-31

**Authors:** Glenn Regnier, Elke Bocksteins, Gerda Van de Vijver, Dirk J. Snyders, Pierre‐Paul van Bogaert

**Affiliations:** ^1^ Laboratory for Molecular Biophysics, Physiology and PharmacologyDepartment of Biomedical SciencesUniversity of Antwerp, CDEAntwerpenBelgium; ^2^ Laboratory for Cardiovascular ResearchInstitute Born‐BungeUniversity of Antwerp, CDEAntwerpenBelgium

**Keywords:** Dorsal root ganglion neurons, Kv2, Kv5‐Kv9, postnatal development

## Abstract

Delayed rectifier voltage‐gated K^+^ (Kv) channels play an important role in the regulation of the electrophysiological properties of neurons. In mouse dorsal root ganglion (DRG) neurons, a large fraction of the delayed rectifier current is carried by both homotetrameric Kv2 channels and heterotetrameric channels consisting of Kv2 and silent Kv (KvS) subunits (i.e., Kv5‐Kv6 and Kv8‐Kv9). However, little is known about the contribution of Kv2‐mediated currents during the postnatal development of DRG neurons. Here, we report that the Stromatoxin‐1 (ScTx)‐sensitive fraction of the total outward K^+^ current (*I*_K_) from mouse DRG neurons gradually decreased (~13%, *P* < 0.05) during the first month of postnatal development. Because ScTx inhibits both Kv2.1‐ and Kv2.2‐mediated currents, this gradual decrease may reflect a decrease in currents containing either subunit. However, the fraction of Kv2.1 antibody‐sensitive current that only reflects the Kv2.1‐mediated currents remained constant during that same period. These results suggested that the fractional contribution of Kv2.2‐mediated currents relative to *I*_K_ decreased with postnatal age. Semiquantitative RT‐PCR analysis indicated that this decrease can be attributed to developmental changes in Kv2.2 expression as the mRNA levels of the Kv2.2 subunit decreased gradually between 1 and 4 weeks of age. In addition, we observed age‐dependent fluctuations in the mRNA levels of the Kv6.3, Kv8.1, Kv9.1, and Kv9.3 subunits. These results support an important role of both Kv2 and KvS subunits in the postnatal maturation of DRG neurons.

## Introduction

Neuronal function depends heavily on the spatial and temporal expression of voltage‐gated K^+^ (Kv) channels which contribute to neuronal excitability by regulating the membrane potential, action potential waveform and firing frequency, transmitter release, and synaptic strength (Hille 2001). Kv channels are integral transmembrane proteins consisting of four *α*‐subunits that form a central ion conducting pore through which K^+^ ions flow according to their electrochemical gradient. Each *α*‐subunit consists of six transmembrane segments (S1‐S6) and a cytoplasmic NH_2_‐ and COOH‐terminus (Long et al. 2005). Based on sequence homology, eight closely *Shaker*‐related subfamilies can be distinguished: Kv1‐Kv6 and Kv8‐Kv9 (Gutman et al. 2005). All members of the Kv1‐Kv4 subfamilies form functional homotetrameric channels and the diversity within these subfamilies is further increased by both the formation of heterotetrameric channels and the interaction with auxiliary *β*‐subunits (Rhodes et al. 1995; Xu et al. 1995). On the other hand, members of the Kv5, Kv6, Kv8, and Kv9 subfamilies do not form functional channels due to retention in the endoplasmic reticulum (ER) and were therefore designated silent Kv (KvS) subunits (for review see (Bocksteins and Snyders 2012)). Co‐assembly of these KvS subunits with members of the Kv2 subfamily relieves this ER retention leading to heterotetrameric Kv2/KvS channel complexes with biophysical properties that differ from the homotetrameric Kv2 channels. These differences include shifts in the voltage dependence of activation and inactivation, changes in gating kinetics, and alteration of the current density.

Due to the molecular diversity of Kv channel complexes, it is very challenging to determine the molecular composition of native Kv currents in neurons. In rodent dorsal root ganglion (DRG) neurons, at least three different Kv currents have been distinguished: the M‐current (*I*_M_), the transient outward current (*I*_A_), and the delayed rectifier current (*I*_DR_) (Akasu and Tokimasa 1992; Gold et al. 1996; Fedulova et al. 1998). The M‐current is a non‐inactivating K^+^ current generated by channels composed of Kv7.2‐Kv7.5 subunits; the major component is carried by heterotetrameric Kv7.2/Kv7.3 channels and homotetrameric Kv7.2 channels (Passmore et al. 2003). *I*_A_ is a very fast activating and inactivating current generated by Kv1.4, Kv3.4, Kv4.1, Kv4.2, and/or Kv4.3 subunits. Depending on the subpopulation of DRG neurons, one or several of these subunits contribute to *I*_A_ (Rasband et al. 2001; Winkelman et al. 2005; Chien et al. 2007; Phuket and Covarrubias 2009). The major component (≈60%) of *I*_DR_ is carried by both homotetrameric Kv2 and heterotetrameric Kv2/KvS channels, although subunits of the Kv1 and Kv3 subfamilies also contribute to *I*_DR_ (Beekwilder et al. 2003; Utsunomiya et al. 2008; Bocksteins et al. 2009, 2012).

The functional diversity of different neuronal subtypes does not only emanate from the molecular composition of different channel complexes but also from the variation in expression of K^+^ currents during different developmental stages (for review see (Ribera and Spitzer 1992)). In the case of the Kv2 subfamily, changes in spatiotemporal expression and cellular abundance have been demonstrated during the development of different neuronal cell types (Maletic‐Savatic et al. 1995; Gurantz et al. 1996; Antonucci et al. 2001; Guan et al. 2011; Sanchez‐Ponce et al. 2012). For example, in rat neocortical pyramidal neurons, the density of Kv2‐mediated currents increased with postnatal age (Guan et al. 2011) while in embryonic *Xenopus* spinal neurons, a Kv2.2‐specific upregulation was demonstrated during maturation (Gurantz et al. 1996). However, it is not known if the contribution of Kv2‐mediated currents to *I*_K_ in DRG neurons is influenced by postnatal age. Therefore, we analyzed the Kv2‐containing currents and characterized the expression of Kv2 and their modulatory KvS subunits in mouse DRG neurons during the first month of postnatal development.

## Material and Methods

### Animals and cell culture

Dorsal root ganglion neurons were obtained from P7 ± 1, P14 ± 1, P21 ± 1, and P28 ± 1 old C57BL/6 male mice. Experiments were conducted in agreement with the European Communities Council Directive on the protection of animals used for experimental and other scientific purposes (2010/63/EU). DRG neurons were isolated as described previously (Schnizler et al. 2008). Briefly, DRGs were dissected from the spinal cord and dissociated by consecutive enzymatic treatment with 2 mg/mL collagenase A (Merck Millipore, Billerica, MA) and 1 mg/mL pronase (Merck Millipore). After enzymatic dissociation, DRG neurons were further dissociated using flame‐polished Pasteur pipettes of decreasing diameters and plated on glass‐bottom dishes coated with poly‐D‐lysine (MatTek Corp., Ashland, MA). Cells were grown in 50:50 DMEM/TNB medium (ThermoFisher Scientific, Waltham, MA/Merck Millipore) supplemented with 2.5% horse serum (ThermoFisher Scientific), 2.5% fetal bovine serum (ThermoFisher Scientific), 100 U/mL penicillin/streptomycin, 1.25% lipid‐protein complex (Merck Millipore), 1 mmol/L l‐glutamine and 0.25 *μ*g/mL nerve growth factor (Sigma‐Aldrich, Saint Louis, MO), and maintained at 37°C in a humidified atmosphere of 5% CO_2_. Electrophysiological and RT‐PCR analyses were performed 3 days after plating.

### Electrophysiology

Whole‐cell patch clamp current recordings were performed on DRG neurons (30–60 pF) at room temperature (20–22°C) with an Axoclamp‐2A amplifier (Molecular Devices, Sunnyvale, CA) in the two‐electrode voltage clamp configuration and were sampled with a TL‐1 labmaster (Molecular Devices). Patch pipettes with a resistance of 3–5 MΩ were pulled from 1.7 mm glass capillaries with a Brown Flaming P‐87 horizontal pipette puller and heat‐polished. DRG neurons were superfused continuously with an extracellular solution, containing (in mmol/L): 140 *N*‐methyl d‐glucamine, 5 KCl, 1 MgCl_2_, 1.8 CaCl_2_, 10 glucose, and 5 HEPES with the pH adjusted to 7.4 with HCl. Pipettes were filled with an intracellular solution, containing (in mmol/L): 140 KCl, 10 HEPES, 5 EGTA, 5 NaCl, 3 MgATP, 1 MgCl_2_, 1 CaCl_2_, and 0.1 cAMP with the pH adjusted to 7.4 with KOH. Outward K^+^ currents were elicited by 500 msec depolarizing pulses between −60 and +60 mV from a holding potential of −70 mV, followed by a 1 sec pulse at −40 mV. Cell capacitance was obtained from the current evoked by a 30 msec step from −60 to −65 mV.

Stromatoxin‐1 (ScTx)‐sensitive currents were obtained by subtracting the currents obtained after application of 300 nmol/L ScTx (Alomone Labs, Jerusalem, Israel) (dissolved in the extracellular solution) from the currents obtained before ScTx application. For the anti‐Kv2.1 current recordings, patch pipettes were dipped in normal intracellular solution and back filled with the anti‐Kv2.1‐containing solution obtained by dissolving 10 *μ*g/mL Kv2.1 antibody (Alomone Labs) in the intracellular solution. Steady‐state reduction of the total outward K^+^ current was reached 15–20 min after patch rupture. The specificity of this reduction (i.e., due to Kv2.1 antibody block and not due to time artifacts) was confirmed previously (Bocksteins et al. 2009). The anti‐Kv2.1‐sensitive currents were obtained by subtracting the currents obtained after steady‐state Kv2.1 block from the currents obtained immediately after patch rupture.

### RT‐PCR analysis

Total RNA was isolated from the DRG cultures as previously described (Bocksteins et al. 2012). Briefly, RNA was isolated using the TriZol (ThermoFisher Scientific) reagent, samples were treated with deoxyribonuclease I (ThermoFisher Scientific) to exclude genomic DNA contamination and cDNA was obtained using the Superscript III RT‐PCR system (ThermoFisher Scientific) according to the manufacturer's guidelines. Expression of the Kv2 and KvS subunits was assessed using gene‐specific primers that spanned intron boundaries (except for the intronless Kv5.1) (Table 1). Glyceraldehyde 3‐phosphate dehydrogenase (G3PDH) was used as a loading control to perform the semiquantitative RT‐PCR analysis. Co‐amplification of the target gene and G3PDH was performed in a reaction mixture containing 1× Colorless GoTaq Flexi buffer, 3 mmol/L MgCl_2_, 0.4 mmol/L dNTP mix, 2.5 U GoTaq G2 Flexi DNA Polymerase (Progema, Madison, WI), and 0.5 *μ*mol/L of each forward and reverse primer. The cDNA samples were amplified for 35 cycles, separated on a 1% agarose gel, and stained with SYBR Safe gel stain (ThermoFisher Scientific) for densitometric analysis. For each PCR analysis, one positive control (reaction that contains the target subunit cDNA) and two negative controls (reaction without cDNA or without reverse transcriptase) were performed. We ensured that the amplification of each gene was still within the exponential phase of the PCR reaction after 35 cycles by comparing the densitometric values with these obtained after 33 and 37 cycles of amplification. Gels were scanned with the LumiImager system using the LumiAnalyst software (Roche Diagnostics, Basel, Switzerland) and the data were analyzed using the ImageJ software (National Institutes of Health, Bethesda, MD). Each PCR product was sequenced to exclude nonspecific amplification.

**Table 1:** List of primer pairs used in semiquantitative RT‐PCR experiments

	Primer pair sequence
G3PDH	5′‐ACGGGAAGCTCACTGGCATG‐3′ 5′‐GGGAGTTGCTGTTGAAGTCG‐3′
K_v_2.1	5′‐TCGACAACACGTGCTGTGCT‐3′ 5′‐GGCCAACTTCAGGATGCGC‐3′
K_v_2.2	5′‐TTGATAACACCTGCTGCCCG‐3′ 5′‐TGGCGAGTTTCAGTATCCTGA‐3′
K_v_5.1	5′‐CTGGTGGGCTATCATCACCA‐3′ 5′‐CGGGTCCTATGATGCTTCTC‐3′
K_v_6.1	5′‐GTCCGTTCTGTTTGTCACCG‐3′ 5′‐GGATCAGCACCCGTTCTTGT‐3′
K_v_6.2	5′‐GGCTCTTCGCCTACGTCTC‐3′ 5′‐CATCACGCGTGCTGTCCTC‐3′
K_v_6.3	5′‐GTGGTGTTCGTGATCGTGTC‐3′ 5′‐CTTGAGAGTCAAGCCCAGTG‐3′
K_v_6.4	5′‐GCCAGGAGTTCTTCTTCGAC‐3′ 5′‐CATCAGGAGACCAAACTCTC‐3′
K_v_8.1	5′‐TCTGCGCATGCTGAAACTGG‐3′ 5′‐AGTACTTGCTCTCTCCCTGC‐3′
K_v_8.2	5′‐CTTCCGAATCCTCAAGCTGG‐3′ 5′‐GTTGACCTTTCCTCGTTCCC‐3′
K_v_9.1	5′‐AGGTAGTGCAAGTGTTCCGC‐3′ 5′‐AAGTCCTCAAACTCGCGCTG‐3′
K_v_9.2	5′‐TTCTCAAGCTGGCCAGACAC‐3′ 5′‐TGACCGAAGGGACCTCTTTC‐3′
K_v_9.3	5′‐TGTAGGGCTTCGGTCTCTTG‐3′ 5′‐AGTACGGTAGCTCATGGCAC‐3′

### Data analysis

Densitometric analysis of the bands obtained after electrophoresis was done using the ImageJ software. The signal intensity of the target band was normalized to the intensity of the G3PDH signal which was represented as relative densitometric value (RDV).

All values are presented as mean ± SEM. Statistical analyses were performed using one‐way ANOVA followed by Tukey's test in case a significant difference was present among the test groups. Trend analysis of age‐dependent changes was performed using linear regression and represented by the regression coefficient ± SE. *P*‐values < 0.05 were considered to be significant.

## Results

### The fractional contribution of the ScTx‐sensitive current gradually decreases during postnatal development while that of the Kv2.1 antibody‐sensitive current remains unchanged

**Figure 1:**
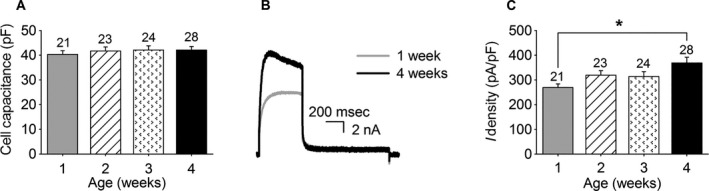
Postnatal development of the whole cell outward K^+^ current in mouse dorsal root ganglion (DRG) neurons. (A) Average cell capacitance of the recorded DRG neurons obtained from 1, 2, 3, and 4 weeks old mice. The numbers above each bar indicate the number of recorded cells. (B) Representative current recordings of the whole cell outward K^+^ current in DRG neurons obtained from 1 week (gray) and 4 weeks (black) old mice elicited by a 500 msec depolarizing pulse to 0 mV from a holding potential of −70 mV. (C) Postnatal development of the whole‐cell outward K^+^ current density at the end of a 500 msec pulse at 0 mV. The current density at 4 weeks was significantly higher than the current density at 1 week, indicated with an asterisk (*P* < 0.05). The numbers above each bar indicate the number of cells analyzed.

To study the potential changes in the Kv2‐mediated currents during the postnatal development of mouse DRG neurons, we determined the fractional contribution of the ScTx‐sensitive and anti‐Kv2.1‐sensitive currents relative to the total outward K^+^ current (*I*_K_) in these neurons obtained from mice at different postnatal ages. We used this dual approach to determine the fractional contribution of the Kv2.1‐ and Kv2.2‐mediated currents relative to *I*_K_ in developing DRG neurons since ScTx inhibits both Kv2.1‐ and Kv2.2‐containing channels (Escoubas et al. 2002) while Kv2.1 antibodies only block Kv2.1‐containing channels (Murakoshi and Trimmer 1999; Guan et al. 2007; Bocksteins et al. 2009). It has previously been demonstrated that the ScTx‐induced Kv2 inhibition is less efficient at higher potentials (>0 mV) (Escoubas et al. 2002) and that the fractional contribution of Kv2‐mediated currents relative to the whole‐cell outward K^+^ current is higher after 500 msec (Bocksteins et al. 2012). Therefore, we determined the fractional contribution of the ScTx‐ and anti‐Kv2.1‐sensitive currents relative to *I*_K_ in the DRG neurons of different developmental stages by normalizing their current density to the total outward K^+^ current density at the end of a 500 msec depolarizing pulse to 0 mV. The ScTx‐ and anti‐Kv2.1‐sensitive currents were obtained as described in Material and Methods, and the current density was determined by dividing the recorded current amplitude at 0 mV after 500 msec by the cell capacitance. For the different development stages, we isolated DRG neurons from P7 ± 1, P14 ± 1, P21 ± 1, and P28 ± 1 mice that were considered as 1, 2, 3, and 4 weeks old mice, respectively. Only DRG neurons with a cell capacitance in the 30–60 pF range were selected for analysis; as a result, the mean whole‐cell capacitance of the analyzed DRG neurons did not change significantly during postnatal development (Fig. 1A). However, the current density of the outward K^+^ current rose gradually (regression coefficient = 30 ± 9 pA/pF/week, *P* < 0.05) with age reaching significance (*P* < 0.05) between 1 week (270 ± 15 pA/pF) and 4 weeks (370 ± 23 pA/pF) (Fig. 1B–C).

**Figure 2:**
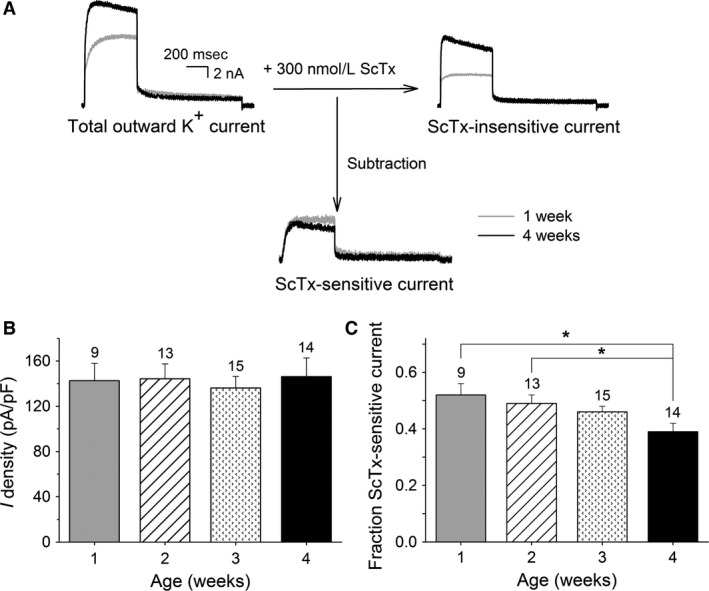
Postnatal development of the ScTx‐sensitive current in dorsal root ganglion (DRG) neurons at 0 mV. (A) Representative current recordings of the total outward K^+^ (left), ScTx‐insensitive (right) and ScTx‐sensitive (bottom) currents in DRG neurons obtained from 1 week (gray) and 4 weeks (black) old mice elicited by a 500 msec depolarizing pulse to 0 mV from a holding potential of −70 mV. The ScTx‐sensitive current was obtained by subtracting the current after application of 300 nmol/L ScTx (i.e., ScTx‐insensitive current) from the total outward K^+^ current. The scale bar applies to all current recordings. (B) Current densities of the ScTx‐sensitive component in the different age groups. The ScTx‐sensitive current density did not change during postnatal development. (C) Fraction of the ScTx‐sensitive current at the different developmental stages obtained by normalizing the current density of the ScTx‐sensitive current to the current density of the total outward K^+^ current at the end of the 500 msec pulse at 0 mV. The fraction of the ScTx‐sensitive current relative to *I*_K_ in DRG neurons from 1 and 2 weeks old mice was significantly larger compared to the same fraction at 4 weeks (*P < 0.05). The numbers above each bar indicate the number of cells analyzed.

Typical current recordings of DRG neurons obtained from 1 and 4 weeks old mice before and after application of 300 nmol/L ScTx are shown in Figure 2A. Extracellular application of 300 nmol/L ScTx reduced *I*_K_ significantly as previously described (Bocksteins et al. 2012). The absolute current density of the ScTx‐sensitive current did not change in DRG neurons of the different age groups (regression coefficient: 0.26 ± 6.39 pA/pF/week, *P* = 0.97) (Fig. 2B): the ScTx‐sensitive current density was 143 ± 15, 144 ± 13, 136 ± 10, and 146 ± 17 pA/pF in DRG neurons of 1, 2, 3, and 4 weeks old mice, respectively. However, the total outward K^+^ current density rose significantly (Fig. 1C) and therefore the fractional contribution of the ScTx‐sensitive current relative to *I*_K_ (FC_ScTx_) decreased gradually (regression coefficient = −0.044 ± 0.012 FC_ScTx_/week, *P* < 0.05) with age reaching significance (*P* < 0.05) at 4 weeks, compared to 1 and 2 weeks old mice (Fig. 2C): ScTx inhibited 52 ± 4% and 49 ± 3% of the outward K^+^ current at week 1 and week 2 respectively, while only inhibiting 39 ± 3% at week 4. These results demonstrated that the fractional contribution of Kv2‐containing channels relative to *I*_K_ in DRG neurons decreased during postnatal development, but these data did not discriminate between Kv2.1‐ and Kv2.2‐containing channels. Therefore, we determined the fraction of anti‐Kv2.1‐sensitive currents.

**Figure 3:**
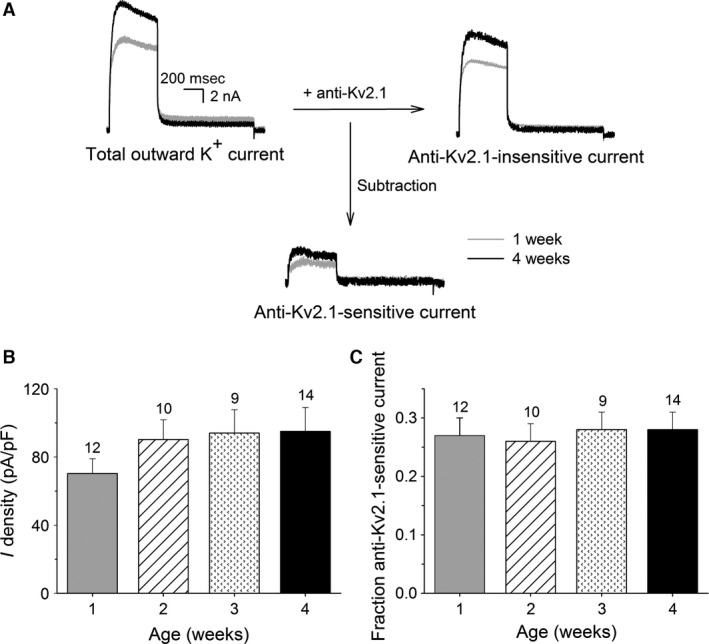
Postnatal development of anti‐Kv2.1‐sensitive current in dorsal root ganglion (DRG) neurons at 0 mV. (A) Representative current recordings of the total outward K^+^ (left), anti‐Kv2.1‐insensitive (right) and anti‐Kv2.1‐sensitive (bottom) currents in DRG neurons obtained from 1 week (gray) and 4 weeks (black) old mice elicited by a 500 msec depolarizing pulse to 0 mV from a holding potential of −70 mV. The anti‐Kv2.1 sensitive current was obtained by subtracting the current after intracellular diffusion of Kv2.1 antibodies (i.e., anti‐Kv2.1‐insensitive current) from the total outward K^+^ current. The scale bar applies to all current recordings. (B) Current densities of the anti‐Kv2.1‐sensitive component in the different age groups. The anti‐Kv2.1‐sensitive current density rose gradually, although not significantly, during postnatal development. (C) The fraction of the anti‐Kv2.1‐sensitive current relative to *I*_K_ at the different developmental stages obtained as described in the Results section remained unchanged. The numbers above each bar indicate the number of cells analyzed.

Representative current recordings of the anti‐Kv2.1‐sensitive currents obtained from 1 and 4 weeks old mice are shown in Figure 3A. Intracellular diffusion of Kv2.1 antibodies reduced *I*_K_ significantly as previously described (Bocksteins et al. 2009). The current density of the anti‐Kv2.1‐sensitive current rose (regression coefficient: 7.7 ± 5.1 pA/pF/week, *P* = 0.14), although not significantly, from 70 ± 9 pA/pF at 1 week to 95 ± 14 pA/pF at 4 weeks (Fig. 3B). However, the fractional contribution of the anti‐Kv2.1‐sensitive current relative to *I*_K_ remained similar in DRG neurons obtained from the different age groups (Fig. 3C): the Kv2.1 antibody blocked 27 ± 3%, 26 ± 3%, 28 ± 3%, and 28 ± 3% of the total outward K^+^ current of the DRG neurons from 1, 2, 3, and 4 weeks old mice, respectively. These results together indicated that the fractional contribution of Kv2.1‐mediated currents relative to *I*_K_ remained similar, whereas the fractional contribution of Kv2.2‐mediated currents relative to *I*_K_ decreased with postnatal age.

**Figure 4:**
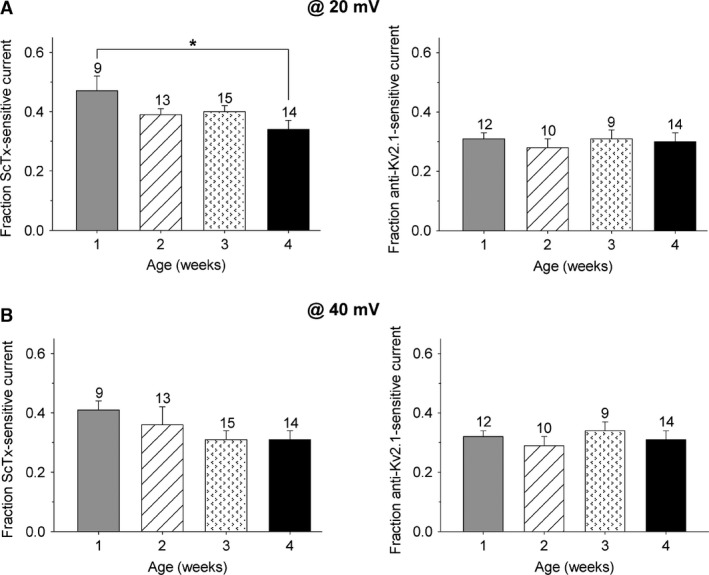
Postnatal development of the ScTx‐ and anti‐Kv2.1‐sensitive current at +20 and +40 mV. The fraction of ScTx‐sensitive current (left) and anti‐Kv2.1‐sensitive current (right) relative to *I*_K_ of the different postnatal age groups at the end of a 500 msec depolarizing pulse at +20 mV (A) and +40 mV (B). (A) At +20 mV, the fraction of ScTx‐sensitive current in dorsal root ganglion neurons from 1 week old mice was significantly larger compared to that from 4 week old mice (*P < 0.05), whereas the fraction of the anti‐Kv2.1‐sensitive current remained unchanged at the different developmental stages. (B) The fraction of ScTx‐sensitive current reduced gradually (although not significantly) with postnatal age at +40 mV, while the fraction of anti‐Kv2.1‐sensitive current at the same potential remained constant during the same period. The numbers above each bar plot indicate the number of cells analyzed.

Since only a fraction of the Kv2.1 and Kv2.2 channels are already in an open state at 0 mV, the above results could be due to an age‐dependent depolarizing shift in the voltage dependence of activation of the Kv2‐containing currents. Therefore, we determined the fraction of ScTx‐sensitive and Kv2.1 antibody‐sensitive current at higher depolarizing potentials (+20 and +40 mV) (Fig. 4). The fraction of the anti‐Kv2.1‐sensitive current remained similar at the different postnatal ages, both at +20 mV (Fig. 4A) and +40 mV (Fig. 4B), whereas the ScTx‐sensitive fraction of *I*_K_ reduced with age at both potentials. At +20 mV, the fractional contribution of the ScTx‐sensitive current relative to *I*_K_ decreased significantly (regression coefficient = −0.036 ± 0.013 FC_ScTx_/week, *P* < 0.05): ScTx inhibited 47 ± 5% at 1 week which is significantly different (*P* < 0.05) from the inhibition at 4 weeks (34 ± 3%) (Fig. 4A). At +40 mV, the fractional contribution relative to *I*_K_ decreased gradually (regression coefficient = −0.035 ± 0.019 FC_ScTx_/week, *P* = 0.075), but not significantly, from 41 ± 3% at 1 week to 31 ± 3% at 4 weeks (Fig. 4B). Although the fractional contribution of the ScTx‐sensitive current relative to *I*_K_ at +20 and +40 mV is lower compared to the fractional contribution at 0 mV due to the incomplete inhibition of the Kv2‐containing currents by ScTx at higher potentials (Escoubas et al. 2002), a comparable decrease with postnatal age could be observed at all analyzed potentials. These results suggested that the observed developmental changes are not due to age‐dependent shifts in the voltage dependence of the Kv2.1‐ and/or Kv2.2‐containing current.

### The Kv2.2 mRNA level decreases during postnatal development while the Kv2.1 mRNA level remains similar

**Figure 5:**
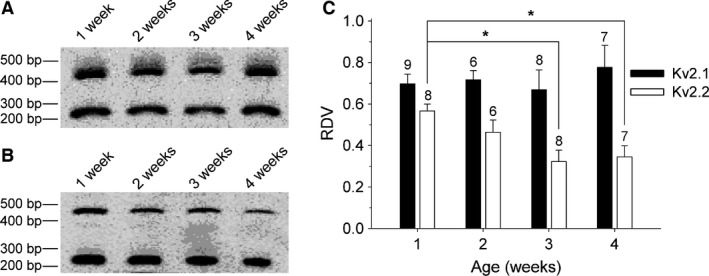
Postnatal development of the Kv2.1 and Kv2.2 mRNA levels in dorsal root ganglion (DRG) cultures. Electrophoretic analysis of the RT‐PCR products obtained from cultured DRG neurons of 1 week, 2 weeks, 3 weeks, and 4 weeks old mice. The relative densitometric values (RDVs) (presented in panel C) were determined by normalizing the densitometric value of the 450 bp fragment, which corresponds to Kv2.1 (A) and Kv2.2 (B), to the densitometric value of G3PDH, which corresponds to the 250 bp fragment in both panels. (C) The RDV of Kv2.2 (white) decreased significantly determined in DRG cultures from 3 and 4 weeks old mice compared to the RDV determined in DRG cultures from 1‐week‐old mice (*P < 0.05). The RDV of Kv2.1 (black) remained unaltered in the different age groups. The numbers above every bar plot indicate the number of samples analyzed.

To investigate whether the observed ScTx‐sensitive and anti‐Kv2.1‐sensitive current densities can be attributed to developmental changes in Kv2.1 and/or Kv2.2 expression, we examined the Kv2.1 and Kv2.2 mRNA levels in our DRG cultures obtained at the different developmental stages using semiquantitative RT‐PCR analysis, as described in Material and Methods. After 35 amplification cycles, the intensity of the Kv signal was normalized to the intensity of the G3PDH signal and plotted as RDV. This semiquantitative RT‐PCR approach revealed that the level of Kv2.2 mRNA decreased gradually (regression coefficient = −0.082 ± 0.022 RDV/week, *P* < 0.05) with age, reaching significance (*P* < 0.05) after 3 weeks: the RDV value declined from 0.57 ± 0.03 at 1 week to 0.32 ± 0.06 and 0.35 ± 0.05 at 3 and 4 weeks, respectively (Fig. 5). On the other hand, the Kv2.1 mRNA level remained similar: the lowest RDV value was detected at 1 week (RDV = 0.70 ± 0.05 RDV/week), while the highest RDV value was detected at 4 weeks (RDV = 0.77 ± 0.11) (Fig. 5). These data indicated that the age‐dependent decrease of the Kv2.2‐mediated current was a result of a developmental decrease of Kv2.2 expression.

### Developmental changes in KvS mRNA levels

It has been demonstrated that different KvS (i.e., Kv5, Kv6, Kv8 and Kv9) subunits are expressed in small mouse cultured DRG neurons (Bocksteins et al. 2009), and both Kv2.1 and Kv2.2 subunits assemble with those KvS subunits into heterotetrameric channels that possess unique biophysical properties (for review see (Bocksteins and Snyders 2012)). Furthermore, at least heterotetrameric Kv2.1/Kv9.3 and Kv2.1/Kv6.3 channels have been shown to be inhibited by ScTx (Escoubas et al. 2002; Moreno‐Dominguez et al. 2009). In addition, we have demonstrated that Kv2.1/Kv9.3 currents are also blocked by Kv2.1 antibodies (Bocksteins et al. 2009). Therefore, it would be interesting to examine whether the observed developmental changes in the ScTx‐ and anti‐Kv2.1‐sensitive currents during the postnatal development of DRG neurons are at least partially due to changes in KvS‐mediated currents. However, no compounds are known yet that can discriminate between Kv2/KvS heterotetramers and Kv2 homotetramers or between different Kv2/KvS channels making it difficult to analyze the up‐ or downregulation of one of the KvS‐containing currents using an electrophysiological approach. Therefore, we used the same semiquantitative approach as described above to investigate the mRNA levels of the different KvS subunits in the different age groups in order to gain a better insight in potential changes that may contribute to the observed developmental changes of the ScTx‐ and anti‐Kv2.1‐sensitive currents.

**Figure 6:**
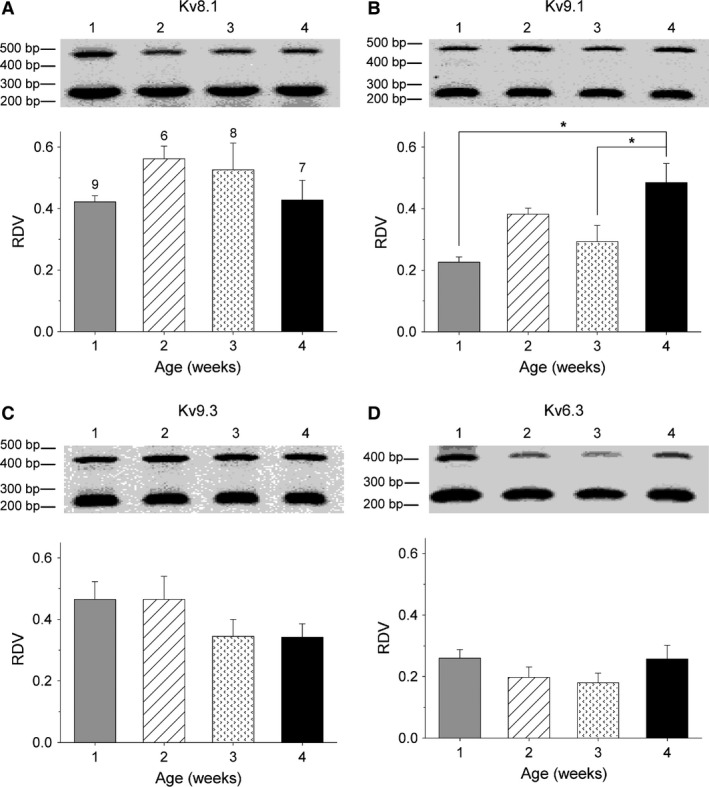
Postnatal development of KvS mRNA levels in dorsal root ganglion (DRG) cultures. Electrophoretic analysis of the RT‐PCR products obtained from cultured DRG neurons of 1, 2, 3, and 4 weeks old mice. The number above each lane corresponds to the age of the mouse represented in weeks. The relative densitometric value (RDVs) of Kv8.1 (A), Kv9.1 (B), Kv9.3 (C), and Kv6.3 (D) were determined by normalizing the densitometric value of the largest fragment, which corresponds to the amplified KvS cDNA, to the densitometric value of the smallest fragment, which corresponds to the amplified G3PDH cDNA. The RDV of Kv9.1 (B) determined in DRG cultures from 4 weeks old mice was significantly higher compared to the RDV determined in DRG cultures from 1 and 3 weeks old mice (*P < 0.05). The numbers above every bar plot in panel A indicate the number of samples analyzed for each age group and are applicable to the other panels.

Similar to our previous study (Bocksteins et al. 2009), the expression of Kv8.1, Kv9.1, and Kv9.3 could readily be observed in our DRG preparations. Furthermore, our semiquantitative approach revealed some age‐dependent variations in the mRNA levels of these KvS subunits which were only significant in the case of Kv9.1 (Fig. 6A–C). The mRNA levels of Kv9.1 displayed an overall increase (regression coefficient = 0.069 ± 0.020 RDV/week, *P* < 0.05), with both 1 week (RDV = 0.23 ± 0.02) and 3 weeks (RDV = 0.29 ± 0.03) being significantly different compared to 4 weeks (RDV = 0.49 ± 0.06).

For Kv8.1, the highest mRNA level was detected at 2 weeks (RDV = 0.56 ± 0.04) and the lowest mRNA level at 1 week (RDV = 0.42 ± 0.02), but these differences did not reach statistical significance. The mRNA levels of Kv9.3 decreased from 1 week (RDV = 0.47 ± 0.06) to 4 weeks (RDV = 0.34 ± 0.04), yielding a regression coefficient of −0.048 ± 0.025 RDV/week (*P* = 0.06). We could also detect Kv6.3 mRNA in our DRG preparations with the highest value at 1 week (RDV = 0.26 ± 0.03) and the lowest at 3 weeks (RDV = 0.18 ± 0.03) (Fig. 6D).

Furthermore, Kv5.1 mRNA appeared to be present in our DRG preparations of every age (Fig. S1A). However, sequence analysis revealed a large nonspecific amplification in these reactions and therefore the relative expression level of the Kv5.1 mRNA could not be determined. The mRNA levels of the other KvS subunits (i.e., Kv6.1, Kv6.2, Kv6.4, Kv8.2, and Kv9.2) could not be detected at any postnatal developmental stage (Fig. S1B–F).

## Discussion

Maturation of different neuronal cell types involves developmental changes in different Kv currents. For example, during the embryonic development of mouse spinal cord neurons, the delayed rectifier K^+^ current appears before the transient outward K^+^ current (Krieger and Sears 1988). In addition, a developmental increase in the whole cell delayed rectifier K^+^ density has been observed in several neuronal cell types (Harris et al. 1988; Surmeier et al. 1991; Spigelman et al. 1992; Martin‐Caraballo and Greer 2000; Falk et al. 2003; Bortone et al. 2006; Guan et al. 2011). These developmental variations in the *I*_DR_ density depend heavily on the spatial and temporal expression of a wide variety of Kv subunits. This is illustrated by changes in the Kv2 expression that have been observed during development of neocortical pyramidal, hippocampal, and spinal neurons (Maletic‐Savatic et al. 1995; Gurantz et al. 1996; Antonucci et al. 2001; Guan et al. 2011; Sanchez‐Ponce et al. 2012). For example, in cultured hippocampal neurons, clustered Kv2.2 expression at the axon initial segment appeared after 10 days in vitro and increased progressively during in vitro maturation, although diffuse expression in the soma and dendrites was detected at all time points (Sanchez‐Ponce et al. 2012). The expression of Kv2.1 and Kv2.2 subunits and their contribution to *I*_DR_ has also been demonstrated in DRG neurons (Ishikawa et al. 1999; Kim et al. 2002; Bocksteins et al. 2009; Tsantoulas et al. 2014). However, it was not known whether the expression of these Kv2 subunits also varies during the postnatal maturation of DRG neurons. In this study, we demonstrated that in DRG neurons, the fraction of ScTx‐sensitive current that represents both Kv2.1‐ and Kv2.2‐mediated currents (Escoubas et al. 2002), decreased with postnatal age while the fraction of anti‐Kv2.1‐sensitive current that only represents Kv2.1‐mediated currents (Murakoshi and Trimmer 1999; Guan et al. 2007; Bocksteins et al. 2009), remained similar (Figs. 2 and 3). These results suggested that the fractional contribution of Kv2.2‐mediated currents relative to *I*_K_ decreased during the postnatal development of DRG neurons which may be caused by a developmental decrease in Kv2.2 expression. Indeed, we detected that the Kv2.2 mRNA level decreased significantly between weeks 1 and 4 (Fig. 5). Since we analyzed the current recordings and RNA samples 72 h after isolation, some changes compared to acute isolation might occur. However, Maletic‐Savatic et al. (1995) observed – in hippocampal neurons – a close spatiotemporal correlation between expression of Kv subunits in situ and during 22 days in vitro culture. Furthermore, the observation that the total outward K^+^ current increases with age in DRG neurons, is consistent with an earlier report (Fedulova et al. 1998). Therefore, we consider it unlikely that major dedifferentiation occurred during the 72 h culture duration, which was the same for each age group.

Although the fractional contribution of the Kv2.1‐mediated current relative to *I*_K_ remained similar with postnatal age, the absolute current density of the Kv2.1‐mediated current increased gradually, but this trend did not reach statistical significance (Fig. 3B). Interestingly, most of the change occurred between weeks 1 and 2. Since there was no increase in Kv2.1 mRNA levels, this may suggest that other factors may modulate the Kv2.1 current density, including post‐translational modifications, association with auxiliary *β*‐subunits and a stimulated translation of the existing mRNA. Various studies have shown a clear correlation between *I*_K_ density and mRNA levels in neurons (Gurantz et al. 1996; Namba et al. 2003; Zhong et al. 2005; Veys et al. 2012), suggesting that this developmental increase in the current density is not likely due to a stimulated translation of mRNA. Changes in post‐translational modifications, including phosphorylation and SUMOylation (Misonou et al. 2004; Plant et al. 2011), and association with the auxiliary *β*‐subunits AMIGO (Peltola et al. 2011), are known to influence the Kv2.1 current density by shifting the voltage dependence of activation of this channel. However, comparison of the fractional contribution of the anti‐Kv2.1‐sensitive current relative to *I*_K_ at higher potentials (>0 mV) indicated that there are no age‐dependent shifts in the voltage dependence of activation of this component (Fig. 4). On the other hand, association of Kv2.1 with other auxiliary *β*‐subunits, including KChAP and KCNE1‐5 (Wible et al. 1998; David et al. 2015), influences the current density of Kv2.1 without affecting the voltage dependence of activation. Consequently, the up‐ or downregulation of *β*‐subunits during postnatal development may contribute to our observed changes in Kv2.1 current density.

The biophysical properties and current density of homotetrameric Kv2 channels are also modulated by the formation of heterotetrameric channels with members of the KvS subfamilies (i.e., Kv5, Kv6, Kv8, and Kv9) (for review see (Bocksteins and Snyders 2012)). Similarly to the Kv2 subunits, the expression of several KvS subunits (and their potential contribution to *I*_K_) in DRG neurons has been demonstrated; we previously detected the expression of Kv6.1, Kv8.1, Kv9.1, Kv9.2, and Kv9.3 in cultured small mouse DRG neurons (Bocksteins et al. 2009) while Tsantoulas et al. (2012) demonstrated the expression of Kv9.1 in myelinated DRG neurons. Here, we confirmed the expression of Kv8.1, Kv9.1, and Kv9.3 subunits in DRG neurons at all postnatal ages (Fig. 6). Interestingly, Kv9.1 expression increased significantly suggesting that Kv9.1 may play an important role in the neonatal maturation of DRG neurons. In contrast, no expression of Kv6.1 and Kv9.2 could be detected at any postnatal stage. This discrepancy could be explained by the difference in RT‐PCR conditions; in our previous study, the expression of these two subunits could only be detected after 44 cycles of cDNA amplification (Bocksteins et al. 2009) while now only 35 amplification cycles were used to avoid potential problems of nonspecific amplifications and to ensure that the amplification of the target gene and G3PDH were both within the exponential phase of the PCR reaction. In addition, we demonstrated the presence of Kv6.3 in the DRG neurons obtained at all postnatal ages while we could not detect any Kv6.3 expression in our previous study. However, in that study, RNA was isolated from dorsal root ganglia of embryonic mice (E12‐E14) (Bocksteins et al. 2009), which suggests that Kv6.3 is not expressed in DRG neurons until the late embryonic and/or neonatal stages.

The total outward K^+^ current density recorded after 500 msec increased during the first month of postnatal development (Fig. 1), while the Kv2‐mediated current density did not change (Fig. 2). This suggests that the density of other delayed rectifier currents such as Kv3‐mediated currents (Bocksteins et al. 2012) and/or M‐currents (Fedulova et al. 1998; Passmore et al. 2003) increases during the postnatal maturation of DRG neurons. Interestingly, in rat DRG neurons, it has been demonstrated that during the first 2 weeks of neonatal life, the current density of *I*_DR_ is decreased while the current density of *I*_M_ increased (Fedulova et al. 1998). These data together may suggest that *I*_DR_ declines during postnatal development due to a decrease in Kv2.2‐mediated currents, while the total outward K^+^ current density increases due to a rise in *I*_M_ current density.

Tsantoulas et al. (2014) demonstrated that inhibiting Kv2‐containing channels in DRG neurons using ScTx resulted in a shortening of the action potential afterhyperpolarization leading to an increased action potential firing rate. However, an induced downregulation of Kv2 in these neurons did not change the duration of afterhyperpolarization which the authors attributed to an altered expression of other ion channels. This indicates that, at least in DRG neurons, a decrease in the Kv2‐containing current either by a decreased Kv2 expression or by blocking the Kv2‐containing current using ScTx does not affect the neuronal properties in the same way. Furthermore, Kv2.1 and Kv2.2 homotetramers produce currents that display similar biophysical properties in heterologous expression systems (Frech et al. 1989; Hwang et al. 1992; Blaine and Ribera 1998). In addition, both Kv2.1 and Kv2.2 subunits can heterotetramerize with members of the silent Kv (KvS) subunits resulting in Kv2/KvS heterotetramers that display distinct properties and that may be affected differently by the use of Kv2‐blocking compounds and/or by changes in Kv2 expression. Taken together, it is difficult to predict the impact of our observed changes on the electrophysiological properties of our DRG neurons. However, the balance between Kv2.1 and Kv2.2 expression changed with postnatal age, suggesting that both subunits predominate (and exert their function) in other stages of the neonatal maturation of DRG neurons.

In conclusion, we propose that the fractional contribution of Kv2.2‐mediated currents relative to *I*_K_ decreases during the postnatal maturation of DRG neurons while the fractional contribution of Kv2.1‐mediated currents remains the same. In addition, the contribution of several KvS‐containing channels fluctuates during the development of DRG neurons. This supports an important role of both Kv2 and KvS subunits in DRG neurons during different developmental stages.

## Supporting information




**Figure S1.** Expression of KvS subunits in adult mouse DRG neurons. RT‐PCR analyses of the Kv5.1 (A), Kv6.1 (B), Kv6.2 (C), Kv6.4 (D), Kv8.2 (E), and Kv9.2 (F) subunits in cultured DRG neurons obtained from 1, 2, 3 and 4 weeks old mice. In each panel, the first lane represents the positive control sample in which the target cDNA was certainly expressed. The second lane represents the first negative control sample in which H_2_O was used instead of cDNA. For each age group, both +RT and −RT samples were tested. The −RT sample represents a negative control whereby the RT reaction was performed without the Reverse Transcriptase enzyme. The +RT sample was used to test the actual expression of the different KvS subunits. No specific amplification of Kv6.1 (B), Kv6.2 (C), Kv6.4 (D), Kv8.2 (E), and Kv9.2 (F) was detected in the DRG samples, whereas the amplification which was detected for Kv5.1 (A) contained a large nonspecific amplification (indicated with an asterisk).
