# Supplementary figures and images for: The contribution of Kv2.2‐mediated currents decreases during the postnatal development of mouse dorsal root ganglion neurons
Source: Physiol Rep. 2016 Mar 31;4(6):e12731. doi: 10.14814/phy2.12731 (PMC4814888; doi:10.14814/phy2.12731)

Figure S1

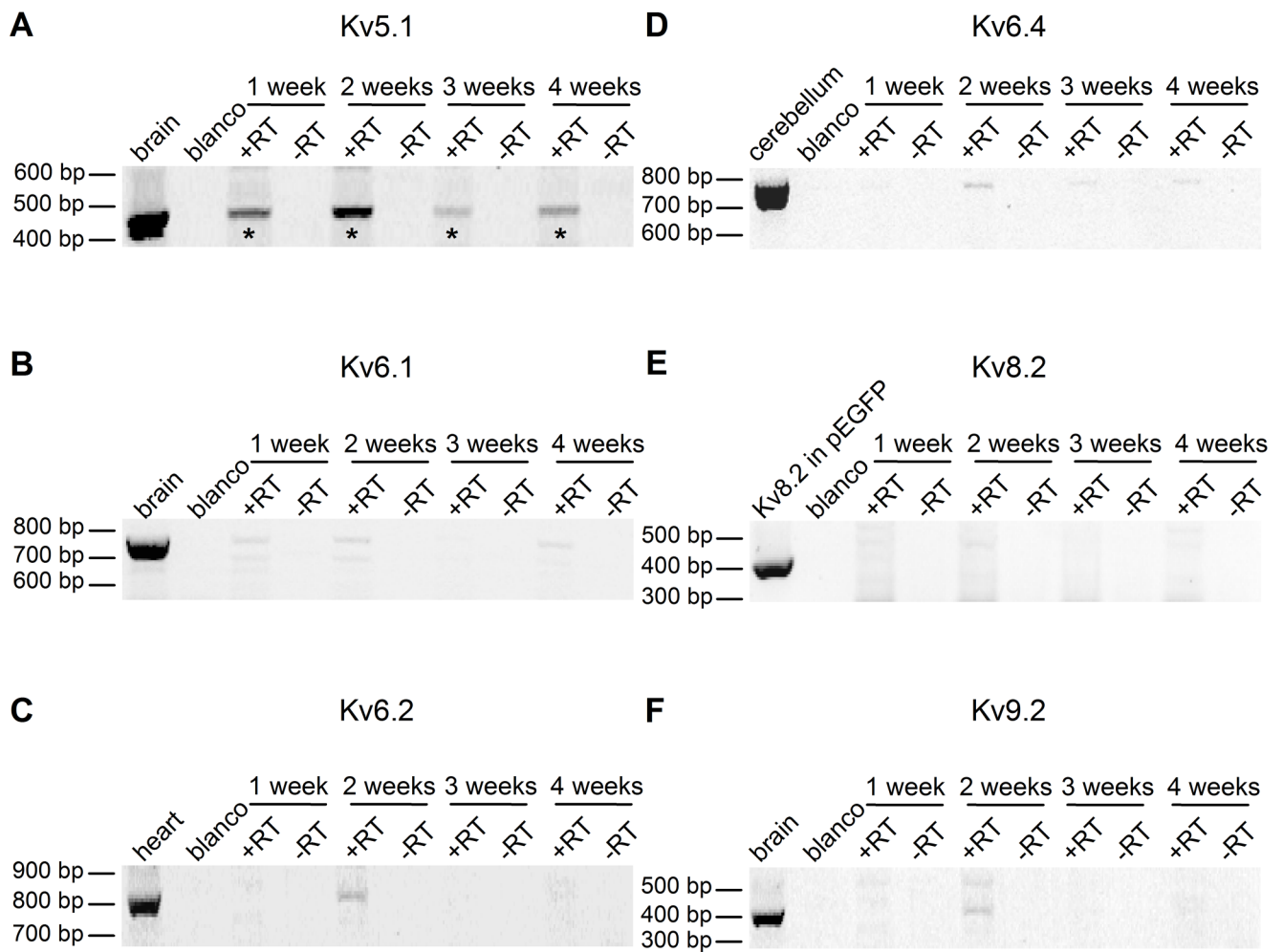

Supplement: Supplementary file 1 — Figure S1. Expression of KvS subunits in adult mouse DRG neurons. RT‐PCR analyses of the Kv5.1 (A), Kv6.1 (B), Kv6.2 (C), Kv6.4 (D), Kv8.2 (E), and Kv9.2 (F) subunits in cultured DRG neurons obtained from 1, 2, 3 and 4 weeks old mice. In each panel, the first lane represents the positive control sample in which the target cDNA was certainly expressed. The second lane represents the first negative control sample in which H2O was used instead of cDNA. For each age group, both +RT and −RT samples were tested. The −RT sample represents a negative control whereby the RT reaction was performed without the Reverse Transcriptase enzyme. The +RT sample was used to test the actual expression of the different KvS subunits. No specific amplification of Kv6.1 (B), Kv6.2 (C), Kv6.4 (D), Kv8.2 (E), and Kv9.2 (F) was detected in the DRG samples, whereas the amplification which was detected for Kv5.1 (A) contained a large nonspecific amplification (indicated with an asterisk). [file PHY2-4-e12731-s001.pdf]
